# How Bioactive Glass S53P4 Kills Bacteria

**DOI:** 10.3390/jfb17040201

**Published:** 2026-04-19

**Authors:** Deeksha Rajkumar, Adrian Stiller, Jurian Wijnheijmer, Ireen M. Schimmel, Leendert W. Hamoen, Leena Hupa, Nicole N. van der Wel, Payal P. S. Balraadjsing, Sebastian A. J. Zaat

**Affiliations:** 1 Department of Medical Microbiology and Infection Prevention, Amsterdam Institute for Immunology and Infectious Diseases, Amsterdam UMC, University of Amsterdam, Meibergdreef 9, 1105 AZ Amsterdam, The Netherlands; p.balraadjsing@amsterdamumc.nl; 2 Laboratory of Molecular Science and Engineering, Åbo Akademi, Henriksgatan 2, 20500 Åbo, Finland; adrian.stiller@abo.fi (A.S.); leena.hupa@abo.fi (L.H.); 3 Swammerdam Institute for Life Sciences, University of Amsterdam, Science Park 904, C3.108, 1098 XH Amsterdam, The Netherlands; j.j.wijnheijmer@uva.nl (J.W.); l.w.hamoen@uva.nl (L.W.H.); 4 Electron Microscopy Centre Amsterdam, Amsterdam University Medical Centre, Meibergdreef 9, 1105 AZ Amsterdam, The Netherlands; i.m.schimmel@amsterdamumc.nl (I.M.S.); n.n.vanderwel@amsterdamumc.nl (N.N.v.d.W.)

**Keywords:** bioactive glass S53P4, antibacterial mechanism, silicon accumulation, membrane disruption, *Staphylococcus aureus*, *Bacillus subtilis*, bacterial cell envelop damage, transcriptomics

## Abstract

Bioactive glass (BAG) S53P4 is a clinically approved bone substitute with antibacterial, osteoconductive and osteostimulatory properties. Its antibacterial effect is associated with ion release, local pH elevation and osmolality, but the precise biochemical and biophysical mode-of-action is unclear. This study investigates the antibacterial mechanism of BAG S53P4 eluates. BAG eluates, collected at 2, 4, 8, and 24 h, eradicated *Staphylococcus aureus*. Elemental analysis revealed an early increase in concentrations of Si and Na, a later rise in Ca, depletion of P over time and rapid loss of Mg. Membrane disturbances occurred within 5 min, evident by permeability for SYTOX, aligning with time-kill kinetics for *S. aureus* and *Bacillus subtilis*. In *B. subtilis*, 2h-BAG-eluate induced rapid delocalization of marker proteins for cell division and DNA repair, signaling membrane potential collapse and nucleoid condensation. Transcriptomics revealed early transcription remodeling reflecting ionic and energetic imbalance, including disruption of central metabolism, redox homeostasis, and translational stability. Scanning electron microscopy revealed severe cell surface damage and particulate deposits on *S. aureus*. Transmission electron microscopy showed cell envelop disruptions and cytoplasmic leakage. Energy dispersive X-ray analysis identified Si on bacterial cell surface at 4 h and intracellular accumulation in punctured, empty cells at 24 h. Overall, BAG ionic dissolution products kill bacteria through a stepwise mechanism involving membrane damage, protein delocalization and metabolic impairment, accompanied by Si deposition on bacterial surfaces and loss of Mg. This finally leads to cell wall degradation, cytoplasmic content leakage and further Si deposition on the cells and inside cell ghosts.

## 1. Introduction

Osteomyelitis-induced bone loss poses a significant clinical challenge due to impaired bone healing, persistent inflammation, and recurrent infection [1]. Standard bone infection management typically involves surgical debridement, bone grafting and prolonged systemic antibiotic therapy. However, these approaches often fail when the infecting bacteria are resistant to antibiotics [2,3]. This highlights the need for alternative therapeutic approaches, such as multifunctional biomaterials that combine local antibacterial effects with osteogenic support to promote bone regeneration at infected defect sites. Bioactive glass S53P4 (abbreviated as BAG in this paper) is a biomaterial composed of 53 weight (wt)% silicon oxide (SiO_2_), 23 wt% sodium oxide (Na_2_O), 20 wt% calcium oxide (CaO) and 4% phosphorus pentoxide (P_2_O_5_) [4]. BAG has been clinically applied as a bone substitute for its combined osteoconductive and antibacterial properties [5]. Upon contact with body fluids, BAG undergoes surface ion exchange with H^+^ followed by gradual network dissolution, leading to the release of Na^+^, Ca^2+^, and phosphate ions, along with soluble silica. These reactions elevate local pH and osmolality. At the glass interface, a silica-rich layer develops, providing nucleation sites for the precipitation of amorphous calcium phosphate, which gradually transforms into a hydroxyapatite-like phase. This mineralized layer supports osteoblast adhesion and bone formation in physiological conditions [6].

In addition to its osteoconductive properties, BAG exhibits broad-spectrum antimicrobial activity against clinically relevant Gram-positive and Gram-negative bacteria as well as fungal pathogens [7,8,9,10], but the mechanism is incompletely understood. Studies on multiple bioactive glass compositions have shown that their antibacterial activity is closely linked to rapid ion release and the resulting rise in environmental pH, supporting an antimicrobial mechanism driven by ion dissolution-induced alkaline and osmotic stress [11,12,13]. While these studies convincingly establish the importance of ion release and alkaline pH for antibacterial effects, they largely correlate dissolution chemistry with bacterial growth inhibition without resolving the fate of the released ions and their role in mediating bacterial cell death, which has remained largely uncharacterized.

In this study, we used an integrated experimental approach to investigate the antibacterial mechanism of BAG. Our investigations started with elemental analysis of BAG eluates by inductively coupled plasma optical emission spectroscopy (ICP-OES), to characterize the elements released from BAG over time. We then performed time-kill and membrane permeabilization (SYTOX) studies to explore the early events of the bactericidal action in *S. aureus*. Complementary experiments with *B. subtilis*, showed responses similar to those of *S. aureus* in time-kill and SYTOX assays, enabling investigation of cellular processes essential for growth, structural maintenance, and viability. To gain deeper insights into the early bacterial response to BAG, transcriptomic analysis was performed. Subsequent microscopic analyses of *S. aureus* using scanning electron microscopy (SEM), transmission electron microscopy (TEM), and scanning transmission electron microscopy-energy dispersive X-ray (STEM-EDX) revealed changes associated with the bactericidal activity of BAG and localized the eluted elements on and inside *S. aureus* bacterial cells.

Our work establishes a connection between the chemical composition of BAG eluates, the influence of the eluted elements on bacterial physiological and transcriptomic responses, and high-resolution imaging of cellular responses and localization of eluted elements in targeted bacteria, offering new insight into the biological mode of action underlying BAG S53P4 antibacterial activity.

## 2. Materials and Methods

### 2.1. Bioactive Glass S53P4

Bioactive Glass (BAG) S53P4 powder (≤25 µm), supplied pre-sterilized by gamma irradiation (Bonalive Ltd., Turku, Finland), was used in this study.

### 2.2. BAG Powder Eluate Collection

BAG powder eluates, produced as described earlier ([10]), were used for the experiments. Briefly, 100 mg of BAG powder was added to wells of a 24-well plate and 1 mL of RPMI 1640 medium (20 mM HEPES, L-glutamine, without sodium bicarbonate; Sigma-Aldrich St. Louis, MO, USA) was added per well to allow elution of ions from the BAG powder for either 2, 4, 8 or 24 h at 37 °C and 120 rpm. Subsequently, the RPMI medium now containing the eluted ions from BAG was collected and centrifuged at 20,800 RCF for 10 min to remove BAG particles. The supernatants were collected and referred to as “2, 4, 8 or 24 h BAG eluate”. Subsequently, the pH was measured using a pH sensor (Eutech Instruments, Singapore; Waterproof pH Testr30). Controls included only RPMI incubated under the same conditions.

### 2.3. Bacteria and Growth Conditions

Inocula of *Staphylococcus aureus* JAR060131 (Culture Collection of Switzerland [CCOS] 890, Wädenswil, Switzerland), originally isolated from a patient with an orthopedic device-related infection [14], were prepared by incubating 1 to 3 colonies in 5 mL Tryptic Soy Broth (TSB; BD Difco, Franklin Lakes, NJ, USA) at 37 °C with shaking at 120 rpm overnight. The following day, 100 µL of the overnight culture was transferred into 5 mL fresh TSB and incubated for 3 h at 37 °C to reach mid-logarithmic growth phase. Cells were washed twice with RPMI and adjusted to 1 × 10^7^ colony forming unit/mL (CFU/mL) in RPMI, based on an established OD-CFU correlation. From this suspension, 100 µL (1 × 10^6^ CFU) or 10 µL (1 × 10^5^ CFU) inocula were used, unless indicated otherwise.

For *Bacillus subtilis*, the wild type strain *B. subtilis* 168 and the following GFP-fusion strains were used to visualize cellular processes: TB35 (*trpC2 amyE::spc–Pxyl–gfp–minD*) to monitor cell division [15] and UG10 (*amyE::spc–Pxyl–recA–mgfp*) to monitor DNA repair. Expression in both strains was induced with 0.5% xylose. Single colonies of each strain were grown overnight in Luria–Bertani (LB; Difco, USA) broth at 30 °C with shaking at 200 rpm, then diluted 1:100 into fresh LB containing 0.5% xylose and grown for approximately 3 h at 30 °C until mid-logarithmic phase (OD_600_ ≈ 0.4; =2 × 10^7^ CFU/mL). Cultures were subsequently adjusted to the inoculum concentrations required for the respective experiments.

### 2.4. Elemental Composition Analysis of BAG Powder Eluate

Concentrations of the elements silicon (Si), sodium (Na), calcium (Ca), phosphorous (P), magnesium (Mg) and potassium (K) in 2, 4, 8 and 24 h BAG eluates and RPMI controls were analyzed using inductively coupled plasma optical emission spectrometry (ICP-OES, Optima 5300 DV; Perkin Elmer, Waltham, MA, USA). For analysis, 375 µL of each eluate was diluted with 10 mL of ultrapure water and acidified with 0.2 mL of concentrated nitric acid (65 wt.%; Suprapur, Merck, Darmstadt, Germany). The instrument was calibrated with ultrapure water and certified standards (all: Spectrascan, Teknolab AS, Norway). Calibration curves were prepared using a dilution series of 0, 1, 5, and 20 ppm for all elements. The following emission lines and configurations were selected: Na (λ = 589.592 nm, radial), Mg (λ = 285.213 nm, axial), Si (λ = 251.611 nm; axial), P (λ = 213.617 nm, axial), K (λ = 766.490 nm, radial), and Ca (λ = 317.933 nm, axial). Instrumental limits of detection (mg/L) were 0.003 (Ca), 0.7 (K), 0.0005 (Mg), 0.025 (P), 0.21 (Na), and 0.004 (Si), corresponding to 0.085, 19.7, 0.014, 0.705, 5.92, and 0.113 mg/L, respectively, in the eluates. For each time point, 5 independent eluates were analyzed. Each eluate was measured using five spectral scans. The reported values of each condition represent the mean of the five independent eluates.

### 2.5. Bactericidal Activity of BAG Powder Eluates

To quantify the bactericidal activity, triplicate samples of BAG eluates were tested undiluted and at 1:2, 1:4, and 1:8 dilutions with RPMI. The bactericidal activity of these eluates against *S. aureus* was evaluated by adding 10 µL of bacterial inoculum (1 × 10^5^ CFU) to 90 µL of each eluate and its dilutions, in a 96-well polypropylene plate (Greiner; cat. 655261). The plates were incubated at 37 °C and 120 rpm for 24 h. Then, these incubations were serially ten-fold diluted, and from each dilution, four 10 µL droplets were plated on blood agar (Oxoid, UK). Plates were incubated overnight at 37 °C and the number of CFU was determined.

### 2.6. Time-Kill and Membrane Permeabilization of BAG Eluate-Treated Bacteria

BAG eluate collected after 2 h (designated as “2h-BAG-eluate” in the remainder of the manuscript) was used for parallel time-kill kinetics and membrane permeabilization assays, both performed with *S. aureus* and *B. subtilis*. Three independent eluates were analyzed.

For time-kill analysis, 10 µL of bacterial inoculum (1 × 10^5^ CFU) was added to 90 µL of each triplicate 2h-BAG-eluate in a 96-well plate and incubated at 37 °C, 120 rpm for 2, 5, 15, 25, 30, 45, 60, 70, 90 min, and 2, 4 and 24 h. At each time point, all incubations were serially diluted and plated on blood agar (BA) to quantify CFU.

Membrane permeability was determined using SYTOX green (Invitrogen), a nucleic acid stain that emits bright green fluorescence upon binding to nucleic acids within cells with permeabilized membranes. A bacterial suspension of 2 × 10^7^ CFU/mL was prepared, from which 5 µL (1 × 10^5^ CFU) was combined with 90 µL of 2h-BAG-eluate and 5 µL of SYTOX green solution (final volume 100 µL). Bacteria with RPMI and heat-killed (HK) bacteria served as negative and positive controls, respectively. Fluorescence (excitation 485 nm; emission 520 nm) was measured every 1.5 min for 240 min at 37 °C using a VANTAstar^®^ plate reader. The time-kill and SYTOX green assay were performed in parallel, each using triplicate measurements, and the correlation between SYTOX green uptake and bactericidal activity was evaluated to determine whether membrane permeabilization correlated with bacterial cell death over time.

### 2.7. Fluorescence Microscopy in 2h-BAG-Eluate Treated B. subtilis

To investigate whether, and which cellular processes would be disrupted by exposure to 2h-BAG-eluate, we used *B. subtilis* strains expressing GFP-fusion proteins that report on cell division (MinD) and DNA repair (RecA). The strains *gfp-minD* (TB35) and *recA-gfp* (UG10) were cultured and induced in 10 mL of Luria’s Broth (LB) at 30 °C as described above. Two ml of culture was harvested at mid-exponential phase (OD_600_ ≈ 0.4), pelleted, and resuspended in an equal volume of 2h-BAG-eluate. Cells were imaged after 5 and 25 min of exposure, using a Nikon Eclipse Ti microscope (Nikon, Tokyo, Japan) with a CFI Plan Apochromat DM Lambda 100× oil objective (NA 1.45, WD 0.13 mm), Hamamatsu ORCA camera (Hamamatsu Photonics K.K., Hamamatsu, Japan), Intensilight HG 130 W lamp, and NIS-Elements AR software. GFP signals were acquired using a GFP filter (Nikon, Tokyo, Japan) set with a 1 s exposure time.

### 2.8. RNA Isolation

RNA isolation protocols were adapted from [16]. Briefly, *B. subtilis* inoculum suspension of 1 × 10^10^ CFU/mL was prepared and 200 µL (1 x 10^9^ CFU) was exposed to 1800 µL of 2h-BAG-eluate for 5 min. After exposure, 500 µL was collected and added to a screw cap tube containing 1.5 g glass beads (0.1 mm), 500 µL phenol/chloroform/isoamyl alcohol mixture (25:24:1) (PCI) and 50 µL 10% SDS, mixed thoroughly and flash frozen in liquid nitrogen. Then, cells were disrupted using a bead-beater (Precellys 24). Phases were separated by centrifugation, whereafter total RNA was precipitated from the aqueous phase using pure ethanol. The precipitated RNA pellet was washed twice with 70% ethanol and dissolved in water. Subsequently, DNA was removed by DNAse I (NEB) treatment. Finally, another round of PCI extraction, ethanol precipitation, washing and elution was done to obtain the final total RNA.

### 2.9. RNA Sequencing and Transcriptomic Data Analysis

RNA samples were treated with RNAse H (NEB) to remove ribosomal RNA species. The RNA-seq library was generated using the NEBNext^®^ Ultra™ II Directional RNA Library Kit from Illumina^®^ (NEB) with NEBNext^®^ Multiplex Oligos for Illumina^®^ (NEB), following the manufacturer’s protocol. Sequencing was conducted using an Illumina NextSeg 550 System with the NextSeq 500/550 Hugh Output v2.5 kit at 75 bp read length. Galaxy was used to process the raw data as follows: trim bad reads and adapter sequences by Trimmomatic, map trimmed reads to the *B. subtilis* reference genome using Bowtie2, count mapped reads using featureCounts and finally quantify differentially expressed features between the samples using Deseq2. The gene-enrichment tool GINtool [17] was used to filter genes, map genes to regulons and generate regulon-level analysis. GINtool uses a priori knowledge on operons, regulons and defined functional categories.

### 2.10. Regulon Analysis

To visualize and prioritize regulatory responses within the transcriptome, a volcano plot was generated at the regulon level using GINtool [17]. In this representation, the average log_2_ fold change in each regulon was plotted on the x-axis, while the statistical significance (log10-transformed *p*-value) was plotted on the y-axis. Regulon-level fold changes and *p*-values were derived from the subset of genes that met the predefined differential expression criteria (*p* ≤ 0.05 and |log_2_FC| ≥ 2), corresponding to at least a 4-fold change in expression in either direction.

Regulon relevance was assessed using the Best Score percentage implemented in GINtool, which reflects the fraction of genes within a regulon that are coherently regulated, i.e., with the regulator either up- or downregulated. This metric allows discrimination between regulons that show consistent, biologically meaningful regulation and those in which only a small subset of genes respond or genes exhibit mixed up and downregulation, resulting in an overall inconsistent pattern.

For the volcano plot, only regulons with a robust relevance score were included, and color-coded according to their relevance category of ≥80% or ≥90%, enabling rapid visual identification of the most strongly and coherently regulated regulons. Together, this approach enabled the extraction of biologically meaningful regulatory patterns from the large number of differentially expressed genes and facilitated the interpretation of the transcriptomic response in terms of coordinated regulatory programs, rather than individual gene changes.

### 2.11. Scanning Electron Microscopy (SEM)

To visualize ultrastructural morphological changes in *S. aureus* after exposure to 2h-BAG-eluate, SEM was performed. An overnight culture of *S. aureus* was grown in tryptic soy broth (TSB) at 37 °C with shaking, and a mid-log culture was obtained by inoculating 800 µL of the overnight culture into 40 mL TSB and incubating for 3 h at 37 °C. The culture was adjusted to 1 × 10^10^ CFU/mL, and 200 µL of this suspension (2 × 10^9^ CFU) was added to 1800 µL of 2h-BAG-eluate or to RPMI control medium. Bacteria were exposed to 2h-BAG-eluate or RPMI control for 24 h at 37 °C, pelleted by centrifugation at 20,800 RCF for 10 min, resuspended in 1 mL EM fixative (4% paraformaldehyde, 1% glutaraldehyde), and fixed for 24 h at 4 °C. Fixed cells were centrifuged again, washed in distilled water for 10 min, and dehydrated in a graded ethanol series (30%, 50%, 70%, 80%, 90%, 96%, 100%, 10 min each) followed by a graded acetone series (30%, 50%, 100%, 10 min each). A drop of poly-L-lysine was placed on glass slides, dehydrated samples were applied, incubated for 5 min and then critical point dried (Leica CPD300) using liquid CO_2_. Bacterial samples were mounted on aluminum SEM stubs, sputter-coated with a thin layer of palladium/platinum (sputtercoater EM Ace600 Leica, Wetzlar, Germany), and imaged using a Zeiss Gemini Sigma 300 field emission gun SEM at 3 kV, using an SE2 detector (Carl Zeiss, Oberkochen, Germany).

### 2.12. Transmission Electron Microscopy (TEM)

Internal ultrastructural changes in *S. aureus* after exposure to 2h-BAG-eluate were investigated using TEM. A total of 200 µL of an *S. aureus* suspension of 1 × 10^10^ CFU/mL was added to 1800 µL of 2h-eluate or to the RPMI control medium and exposed for 4 or 24 h at 37 °C, 120 rpm. Cells were pelleted (20,800 rcf, 10 min), resuspended in EM fixative, and subsequently transferred into storage buffer (0.1 M PHEM, 0.5% paraformaldehyde). Samples were stored at 4 °C until further processing.

Fixed bacteria in storage buffer were washed with H_2_O, centrifuged at 2640 RCF for 5 min and incubated for 1 h in osmium tetroxide (1% in ddH_2_O). After this, the bacteria were washed with H_2_O for 15 min and dehydrated with increasing concentrations of EtOH for 15 min per concentration (70%, 70%, 80%, 90%, 90%, 96%, 100%, 100%). After each step, bacteria were centrifuged at 5000 rpm for 5 min, and the supernatant was removed. Then, bacteria were infiltrated for 2 h with 1:1 epon (electron microscopy sciences) and propylene oxide. After centrifugation at 7000 rpm for 10 min, the upper phase formed by the viscosity-driven epon-propylene oxide gradient was removed. Fresh epon was added and infiltrated. Lastly, the epon was refreshed, and the samples were polymerized at 60 °C for at least 2 days. The samples were cut using a diamond knife (Diatome) using a Leica Ultracut UC7 microtome (Leica, Wetzlar, Germany), and sections were contrasted with uranyl acetate (3.5% in ddH2O) and lead citrate (Electron Microscopy Sciences, cat #22410). The sections were imaged using a Talos L120c 120 kV microscope with a BM-Ceta camera (Thermo Fisher Scientific, Waltham, MA, USA).

### 2.13. Energy Dispersive X-Ray Analysis (EDX)

To determine elemental distribution, *S. aureus* was exposed for 4 h and 24 h to 2h-BAG-eluate, processed for TEM (as above), and analyzed by EDX on a 100–150 nm ultrathin contrasted section (as described above). Sections were then coated with 15 nm carbon using a Leica ACE600 sputter coater (Leica Microsystems, Wetzlar, Germany). EDX analysis was performed using a Talos L120c transmission electron microscope (Thermo Fisher Scientific, Waltham, MA, USA) at 120 kV in scanning transmission electron microscopy (STEM) mode at a 15° angle tilted towards the Bruker XFlash 6T detector (Bruker, Billerica, MA, USA). Data was acquired using Esprit 2.2 software with an acquisition time of approximately 1 h per image and analyzed using a standardless Cliff-Lorimer method combined with Bayes deconvolution. The resulting data were used to generate spectra of a broad array of elements and matching illustrative STEM-EDX images showing the localization of selected elements.

### 2.14. Statistics

Statistical analysis was performed using GraphPad Prism 10 (GraphPad, San Diego, CA, USA). Bactericidal activity was analyzed by two-way ANOVA with Tukey’s post hoc test. A *p*-value ≤ 0.05 was considered significant.

## 3. Results

### 3.1. Elemental Analysis of BAG Powder Eluates

**Figure 1:**
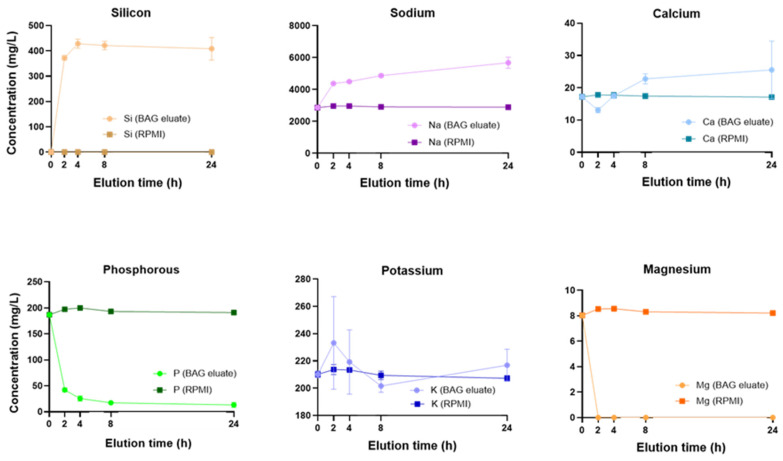
Elemental profiles from BAG eluates at 2, 4, 8 and 24 h, measured using ICP-OES and compared to RPMI controls. From left to right, concentrations (mg/L) of Si, Na, Ca, P, K and Mg over time. Values represent mean ± SD.

BAG powder eluates collected at 2, 4, 8, and 24 h were analyzed for their elemental composition using ICP-OES. The concentrations of Si, Na, Ca, P, K, and Mg in the BAG eluates were quantified and compared with the concentrations in RPMI controls (Figure 1).

In BAG eluates, Si concentrations rose from 0 to ~372 mg/L in 2 h and to ~428 mg/L at 4 h and then remained relatively stable, ranging from 410 to 430 mg/L at later time points. As expected, Si was not detected in the RPMI controls. Na concentrations increased rapidly from ~3000 mg/L in the RPMI controls to ~4500 mg/L within 2 h and continued to rise steadily, reaching ~6000 mg/L at 24 h. Ca concentrations initially decreased from ~17 to ~13 mg/L at 2 h relative to RPMI, then increased to ~17 and ~25 mg/L at 4 and 24 h, respectively. P concentrations in BAG eluates were consistently and markedly lower than in RPMI controls, decreasing from an initial value of ~197 to ~42 mg/L at 2 h to ~13 mg/L by 24 h. K concentrations fluctuated around the RPMI control values across all time points, although replicate variability was high. Mg was depleted below detection in BAG eluates at all time points. Overall, BAG eluates showed marked Si release, Na and Ca elevation, P depletion, and complete Mg loss from the RPMI medium over time.

### 3.2. Bactericidal Activity of BAG Powder Eluates

**Figure 2:**
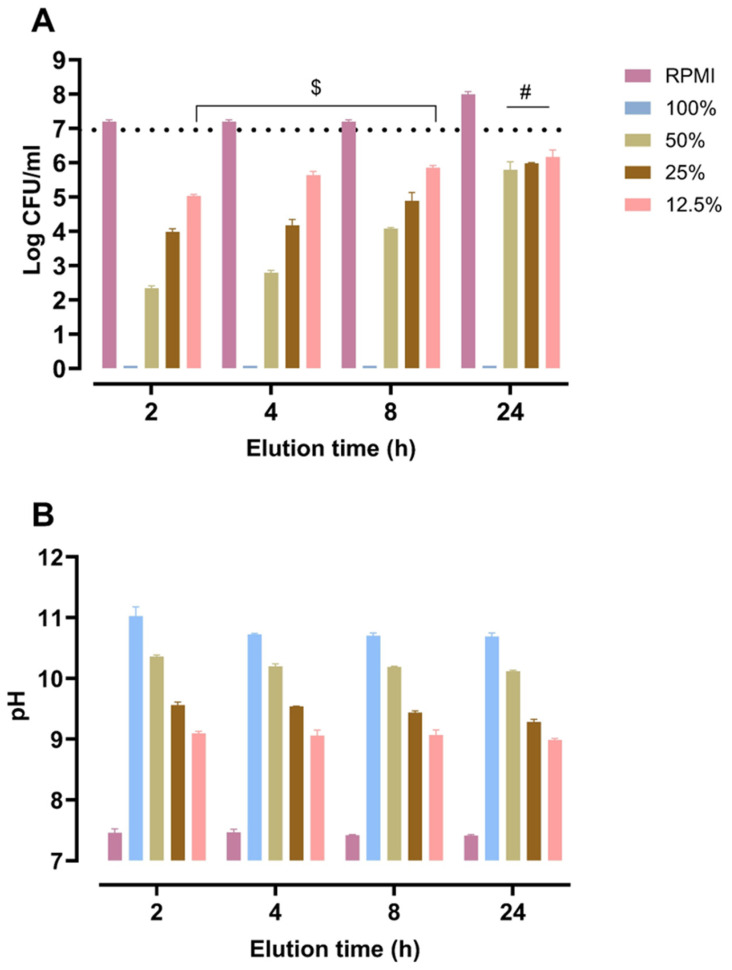
Bactericidal activity and pH of BAG eluates collected at 2, 4, 8, and 24 h. (**A**) Bactericidal activity of undiluted (100%) and diluted (50%, 25%, 12.5%) eluates against *S. aureus*. The dotted line indicates the initial inoculum; data represent mean ± SD. Undiluted eluates (100%) showed complete bacterial killing with no differences between time points. At 50%, 25% and 12.5% eluates collected at 2, 4, and 8 h showed similar activity except for a slight decrease in activity for the 12.5% diluted 8 h eluate compared to the 2 h eluate ($, *p* ≤ 0.05). The 24 h eluate showed significantly lower activity than 2, 4, and 8 h eluates for all respective dilutions (#, *p* ≤ 0.05). Exact *p*-values for all comparisons are provided in Table S1. (**B**) Corresponding pH values of BAG eluates and their dilutions, bars indicate mean + SD.

BAG eluates collected at 2, 4, 8, and 24 h were tested for bactericidal activity in undiluted form (100%) and at 50%, 25%, and 12.5% dilutions. All undiluted eluates caused the complete killing of the *S. aureus* inoculum. Upon dilution, differences in activity of the eluates collected at different time points became apparent. Eluates collected at the earliest time points were most active, and eluates collected at 24 h were least active, losing their bactericidal activity even upon two-fold dilution (Figure 2A).

The pH of the 2 h eluate was highest, 11.0, slightly dropped at 4 h elution to 10.6 and remained stably alkaline across the later elution time points for the undiluted (100%) eluates (Figure 2B). Dilution of the eluates to 50%, 25%, and 12.5% progressively lowered pH values, showing a relationship between bactericidal activity and alkalinity.

Although the 50% dilutions of the 2 h and 24 h eluates showed comparable pH values, the 24 h eluate exhibited a markedly lower bactericidal effect. This suggests that bactericidal activity is not governed by pH alone. Since the early eluates exerted superior bactericidal activity compared to 24 h eluates, the undiluted 2 h eluate was selected for further experiments. This eluate is further referred to as “2h-BAG-eluate”.

### 3.3. 2h-BAG-Eluate Exerts Rapid Bactericidal Activity by Compromising the Bacterial Membrane

To enable more mechanistic studies, we introduced *B. subtilis* as a complementary model organism. The availability of strain expressing gfp-fused fluorescent marker proteins allowed sensitive detection of early physiological changes and to correlate those with gene expression responses following 2h-BAG-eluate exposure. Moreover, as a Gram-positive bacterium with a broadly comparable cell envelope architecture and core metabolic features to *S. aureus*, *B. subtilis* provides a relevant model system for probing the membrane effects of antimicrobial agents under well-controlled experimental conditions [18].

**Figure 3:**
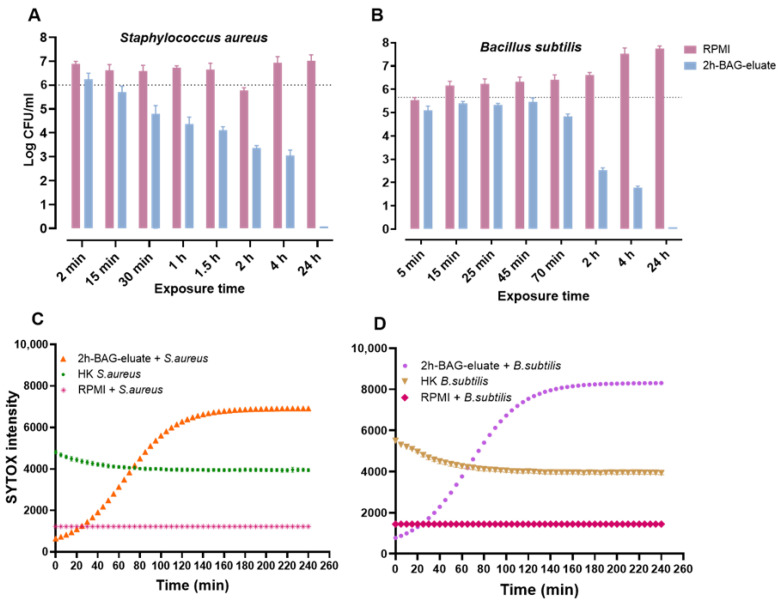
Time-kill kinetics and membrane permeabilization of *S. aureus* and *B. subtilis* exposed to 2h-BAG-eluate). (**A**) Time-kill kinetics of *S. aureus* and (**B**) *B. subtilis* exposed to 2h-BAG-eluate Bars indicate mean + SD and the dotted line represents the bacterial inoculum. (**C**) SYTOX green fluorescence intensity over time in *S. aureus* and (**D**) *B. subtilis*. Heat-killed (HK) bacteria represent maximum membrane permeabilization, while RPMI + bacteria serve as the intact control.

To determine the speed of bacterial killing, a time-kill assay using 2h-BAG-eluate was performed against *S. aureus* and *B. subtilis*. *S. aureus* exhibited a ~1-log CFU reduction within 30 min, followed by a gradual decline in CFU and complete eradication by 24 h (Figure 3A). *B. subtilis* showed a ~1-log CFU reduction at 70 min, after which a rapid decline started at 2 h and complete killing by 24 h (Figure 3B). These findings indicate that while both species are sensitive to 2h-BAG-eluate, *B. subtilis* requires a longer initial exposure before CFU reduction starts, after which the reduction in CFU numbers proceeds more rapidly.

To examine the mechanism underlying this rapid killing by the 2h-BAG-eluate, in parallel to the time-kill assay, membrane permeabilization was evaluated using the SYTOX green fluorescent stain. SYTOX fluorescence intensity increased in both *S. aureus* (Figure 3C) and *B. subtilis* (Figure 3D) from ~5 min onward, indicating rapid cell membrane permeabilization. SYTOX fluorescence rose steadily and plateaued at 140 min, concurrent with decreasing numbers of CFU. Heat-killed controls showed a rapid development of fluorescence followed by a slight decline, whereas control cells in RPMI medium did not exhibit fluorescence. Taken together, these results demonstrate that 2h-BAG-eluate rapidly initiated permeabilization of the bacterial cytoplasmic membrane and that the process gradually led to killing of the entire inoculum.

### 3.4. 2h-BAG-Eluate Rapidly Disrupts Membrane Potential and Membrane Barrier Integrity in B. subtilis

**Figure 4:**
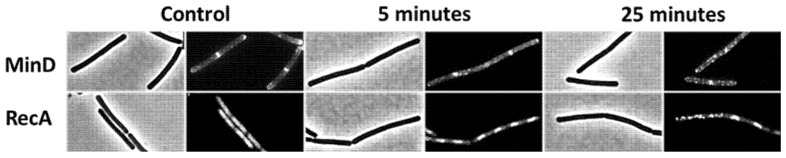
Delocalization of MinD and condensation of RecA following exposure to 2h-BAG-eluate. *B. subtilis* cells expressing MinD (**upper row**) and RecA reporters (**lower row**) before (control) and after 5 or 25 min of exposure to 2h-BAG-eluate. Phase contrast (**left**) and fluorescence images (**right**) are shown for each condition. Frame width is 20 micrometers.

To investigate cellular processes fundamental for growth, structural integrity, and survival, *B. subtilis* was exposed to 2h-BAG-eluate. Within 5 min of exposure, we observed the gradual delocalization of cell division protein MinD (Figure 4). The peripheral membrane protein MinD requires the presence of an intact membrane potential to localize correctly [19] and its delocalization therefore suggests disruption of the membrane potential.

Consistent with disruption of membrane function, DNA repair protein RecA showed increased condensation after 5 min of exposure (Figure 4). RecA localizes to the nucleoid, where it binds single-stranded DNA and mediates homologous recombination during DNA repair [20]. The observed RecA condensation, therefore, suggests nucleoid condensation, a phenotype frequently associated with protoplast shrinkage following the leakage of intracellular contents [15,21]. After 25 min of exposure, both RecA and MinD localization became completely aberrant, consistent with severe disruption and likely secondary effects. Together, these observations suggest that 2h-BAG-eluate rapidly disrupts membrane potential and compromises membrane barrier function.

### 3.5. Transcriptomic Response of Bacillus subtilis to 2h-BAG-Eluate

To further characterize the early bacterial response to BAG, we performed transcriptomic analysis of *B. subtilis* following 5 min exposure to the 2h-BAG-eluate.

Transcriptome analysis revealed extensive differential gene expression in response to 2h-BAG-eluate exposure. Log_2_ fold changes (log_2_FC) were calculated relative to the untreated control, such that positive values indicate upregulation upon BAG exposure and negative values indicate downregulation. In total, 555 operons were significantly differentially expressed (*p* ≤ 0.05 and |log_2_FC| ≥2) (Table S2), indicating rapid and broad transcriptional remodeling. To extract biologically meaningful regulatory patterns, we performed a regulon-level analysis focusing on inferred regulatory activity rather than individual gene fold-changes.

Genes meeting the differential expression criteria were grouped according to known regulons [22], and for each regulon (i) the direction of the change in expression of each gene was adjusted according to whether the regulator activates or represses that gene, (“signed”); (ii) average signed fold change was calculated, representing inferred regulator activity and (iii) a relevance score (percentage of coherently regulated genes changed in expression level) was used to assess regulon coherence [17] (Table S3).

**Figure 5:**
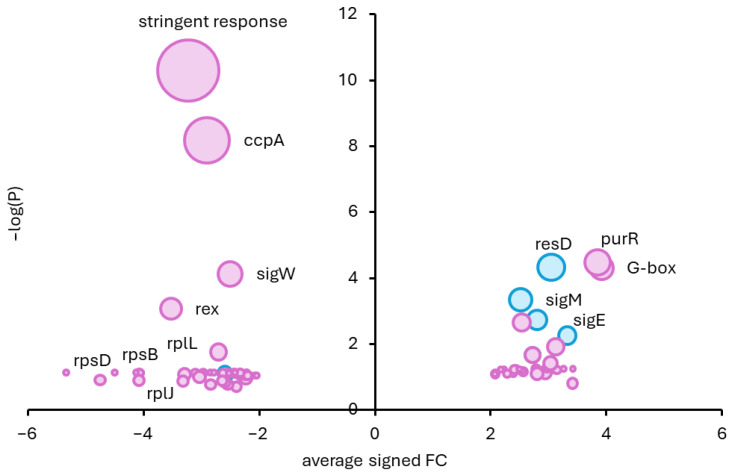
Regulon-level volcano plot of *B. subtilis* transcriptional response to 2h-BAG-eluate. Average signed log_2_ fold change (x-axis) and statistical significance (−log_10_ *p*-value, y-axis) of regulons, calculated from significantly differentially expressed genes (*p* < 0.05, |log_2_FC| ≥ 2; Table S2). Log_2_FC values are defined relative to the untreated control, with positive values indicating upregulation upon BAG exposure and negative values indicating downregulation. Only regulons with high internal coherence (≥80% relevance) are shown. Bubble size = regulon size (Nr Genes); blue/purple = relevance score ≥80%/≥90%.

**Figure 6:**
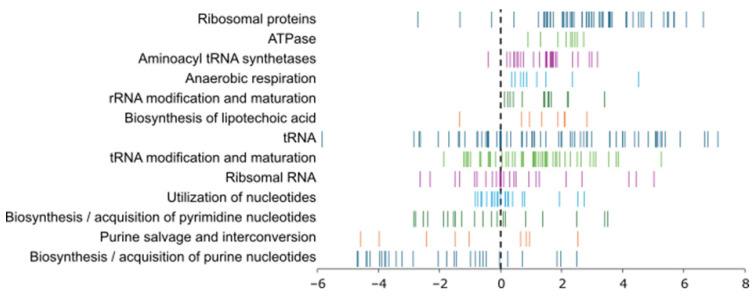
Log_2_FC values (x-axis) of individual genes classed to biological function categories. Log_2_FC values are defined relative to the untreated control, with positive values indicating upregulation upon BAG exposure and negative values indicating downregulation.

Among the most significantly affected regulons was the PurR regulon. PurR acts as a transcriptional repressor that downregulates genes involved in *de novo* purine biosynthesis. In response to the 2h-BAG-eluate, PurR activity increased (Figure 5), leading to strong repression of its target genes such as *purF* and *purI* (Table S2). Despite this repression, in the regulon analysis framework, this appears as a positive signed regulon score, since expression of the regulator is increased (Figure 5). This response suggests rapid suppression of nucleotide biosynthesis (Figure 6), consistent with an early disengagement from growth-related metabolic programs following exposure to 2h-BAG-eluate.

Following 2h-BAG-eluate exposure, genes associated with central carbon metabolism were strongly affected. The CcpA regulon showed a pronounced negative shift in the regulon-level volcano plot (Figure 5), indicating reduced inferred CcpA activity. CcpA is a global carbon catabolite regulator that coordinates the use of preferred carbon sources by modulating transcription through promoter architecture and *cre*-site (catabolite response element) positioning [23]. The negative CcpA regulon fold-change was driven by downregulation of genes normally activated by CcpA, and by derepression of CcpA-repressed targets. This pattern is consistent with functional inactivation of CcpA-dependent carbon catabolite control. This response reflects the rapid disturbance of energy metabolism and intracellular metabolite pools following membrane damage [24]. Under such conditions, carbon flux and glycolytic/overflow pathways are commonly rebalanced as cells attempt to maintain ATP generation and redox homeostasis despite impaired respiratory efficiency [24]. Given that 2h-BAG-eluate has a high pH, the observed CcpA regulon expression shift may additionally reflect altered intracellular metabolite and redox states caused by alkaline stress, which is known to perturb carbon catabolite control and glycolytic flux independently of nutrient availability [25,26]

A prominent transcriptional signature revealed by the volcano plot involved redox and respiratory control. Rex and ResD serve as intertwined transcriptional regulators of redox and respiratory metabolism in Gram-positive bacteria such as *B. subtilis* and *S. aureus*. Rex monitors the NADH/NAD^+^ ratio to repress fermentation and anaerobic genes under high-oxygen conditions, while ResD acts as a two-component system activator that promotes the shift to anaerobic respiration (e.g., via nitrate or nitrite utilization) [27,28,29,30]. Rex is a redox-sensitive transcriptional repressor that dissociates from DNA upon NADH accumulation. Upregulation of its target genes, therefore, indicates functional inactivation of Rex due to an elevated intracellular NADH/NAD^+^ ratio. In our dataset, the Rex regulon was strongly repressed (Figure 5), leading to derepression of Rex-controlled genes such as *ndh* and *cydABC (log_2_FC of 2.99, 6.24 and 5.38)* (Table S2). The observed expression pattern provides strong evidence for redox imbalance and impaired electron transport shortly after 2h-BAG-eluate exposure. Notably, induction of the *cyd* genes mentioned above has also been observed under alkaline conditions [25], suggesting that the respiratory remodeling observed here likely results from the combined effects of membrane disruption and extracellular alkalinization. Consistent with this interpretation, the ResD regulon, which controls genes involved in respiratory adaptation [31,32], was significantly induced (Figure 5). Because ResD functions as a transcriptional activator, upregulation of its target genes indicates increased ResD activity. Activation of ResD-regulated pathways, therefore, suggests compensatory transcriptional remodeling of respiratory processes in response to compromised electron transport. Together, the Rex derepression and ResD activation define a coordinated response to respiratory dysfunction and redox stress, consistent with the membrane damage observed in SYTOX uptake assays (Figure 3C,D).

Cell envelope-associated stress responses were differentially modulated. The SigM and SigE regulons were induced (Figure 5 and Table S3), as evidenced by upregulation of genes positively controlled by these sigma factors, indicating activation of cell envelope stress responses [33,34,35,36]. In contrast, the SigW regulon was significantly repressed. The SigW regulon is typically activated in response to antimicrobial peptides [37] and alkali shock [38] through a regulated, membrane-embedded signaling cascade. As activation of SigW requires an intact membrane-embedded anti-sigma factor and regulated intramembrane proteolysis [39], repression of the SigW regulon under these conditions can be explained by disruption of membrane-dependent signal transduction. Together, this regulatory pattern indicates that exposure to 2h-BAG-eluate affects cell envelope-associated stress responses in a manner distinct from canonical sensor-driven activation.

Among the most strongly upregulated genes following 2h-BAG-eluate exposure were genes encoding components of the translational machinery, including ribosomal proteins, ribosome stability and assembly factors, elongation factors, and multiple aminoacyl-tRNA synthetases (Figure 6) (Table S2). Several ribosomal proteins encoded by genes such as *rpsD*, *rpsB*, *rplJ* and *rplL*, function as auto-repressors and appear as single-gene repressed regulons (Figure 5). This repression pattern extends beyond structural ribosomal genes and indicates a broader downregulation of the protein synthesis apparatus. Ribosome structure and function are highly sensitive to intracellular ionic conditions, particularly to magnesium concentrations. Under ionic imbalance or reduced magnesium availability, ribosomal subunits are known to dissociate and translation efficiency declines [40,41,42,43]. The observed upregulation of genes involved in translation likely represents a compensatory transcriptional response to functional ribosome destabilization, intended to maintain translational capacity despite ribosome instability.

Overall, the early transcriptional response to 5 min of exposure to 2h-BAG-eluate reflects immediate physiological disruption driven by membrane permeabilization, ionic imbalance, and redox collapse. The combined signature of PurR activation, CcpA inactivation, Rex derepression, ResD activation, selective sigma factor modulation, and compensatory induction of translational machinery defines a coherent stress landscape consistent with rapid impairment of membrane integrity and respiratory function.

### 3.6. Ultrastructural Damage of S. aureus Induced by 2h-BAG-Eluate

#### 3.6.1. SEM

**Figure 7:**
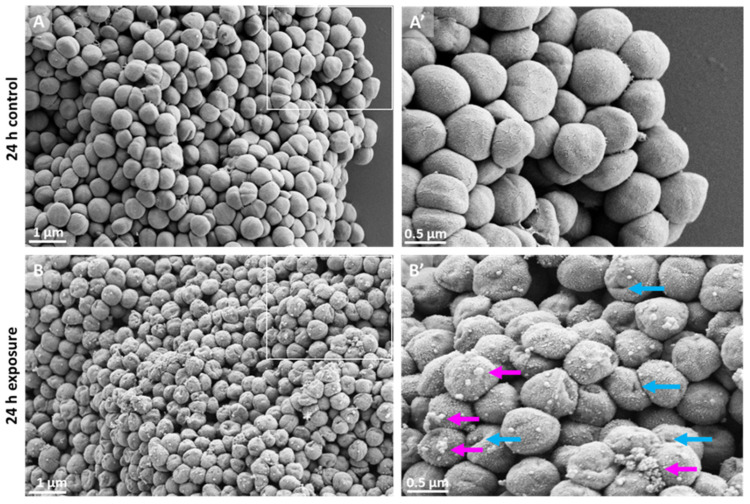
SEM micrographs of *S. aureus* showing intact bacterial cells in the control samples (**A**,**A’**). After exposure to 2h-BAG-eluate for 24 h (**B**,**B’**), bacterial cells’ morphology is altered with shape irregularities and holes (blue arrows) and accumulation of particles on the surface (pink arrows), boxed region enlarged (in (**B’**)). White bars indicate scale.

To visualize potential late-stage ultrastructural alterations, *S. aureus* was exposed to 2h-BAG-eluate for 24 h and examined by SEM. In the RPMI control, *S. aureus* cells exhibited smooth, spherical cocci with intact cell envelopes forming densely packed aggregates (Figure 7A,A’). Following 24 h exposure to the 2h-BAG-eluate, bacterial cells exhibited altered morphology, including irregular contours and holes, as well as the presence of particulate deposits on the cell surface (Figure 7B,B’).

#### 3.6.2. TEM

**Figure 8:**
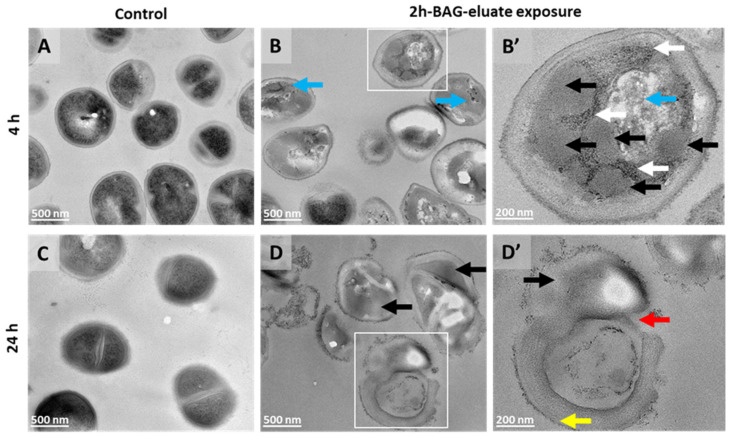
TEM micrographs of *S. aureus* controls and exposed to 2h-BAG-eluate for 4 and 24 h. Control samples show intact bacterial cells (**A**,**C**). After exposure to 2h-BAG-eluate for 4 h (**B**,**B’**) and 24 h (**D**,**D’**), bacterial cells begin to break down and fall apart. Blue arrows indicate DNA, black arrows likely indicate lipid inclusions, white arrows indicate ribosomes, the yellow arrow indicates an electron-dense layer attached to the cell wall and the red arrow indicates disruption of the cell wall. Electron-lucent areas are areas where material has been lost during sample preparation. Scale is 500 nm (**A**–**D**) and 200 nm (**B’**,**D’**).

To assess the effects of 2h-BAG-eluate on *S. aureus* (intra)cellular structural integrity in more detail, TEM analysis was performed after 4 h and 24 h of exposure to 2h-BAG-eluate. In controls, cells exhibited intact morphology with well-defined envelopes and uniform cytoplasmic density and ribosome distribution, indicative of metabolically active cells (Figure 8A,C). In contrast, 2h-BAG-eluate-exposed bacteria displayed progressive structural alterations after 4 h. Upon exposure, cells exhibited irregular morphology, with ribosome-free regions (black arrows) and their DNA disrupted or condensed (blue arrows in Figure 8B,B’). After 24 h, severe structural disruption was evident, with loss of cell wall integrity causing holes (red arrow), leakage or complete absence of cytoplasmic content and presence of extracellular debris (Figure 8D,D’). In addition, some bacteria had a thickened cell wall and an electron-dense ruffled coat (yellow arrow) (Figure 8D’), suggesting aberrant cell wall synthesis or structure maintenance.

### 3.7. Time-Dependent Interaction of BAG-Derived Ions with S. aureus Detected by EDX Analysis

**Figure 9:**
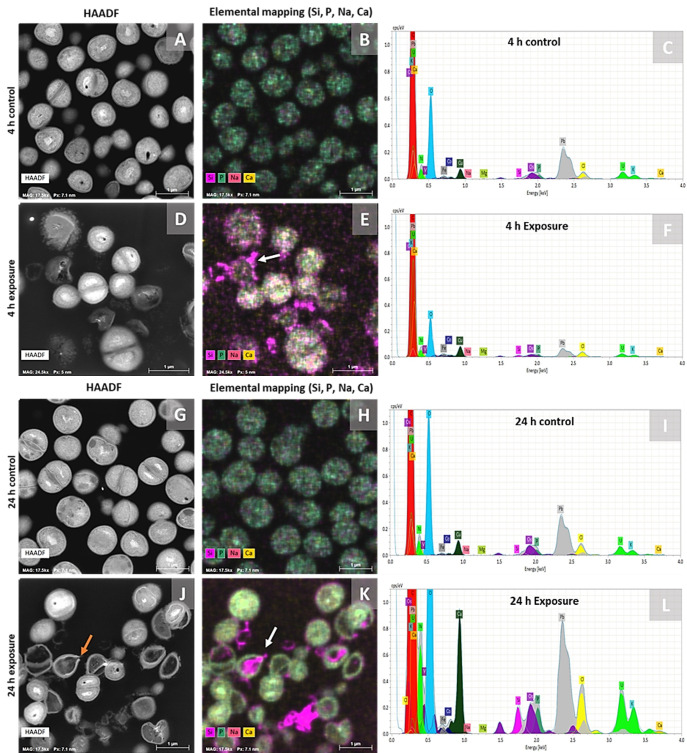
STEM-EDX analysis of *S. aureus* after 4 h and 24 h exposure to the 2 h-eluate. (**A**–**C**) 4 h control: (**A**) HAADF (**B**) EDX elemental images for Si, P, Na, Ca and (**C**) spectrum. (**D**–**F**) 4 h exposure: (**D**) HAADF (**E**) EDX images revealing Si particles at cell wall surface (white arrow), diffuse intracellular Ca and P, (**F**) spectrum. (**G**–**I**) 24 h control: (**G**) HAADF (H) EDX images (**I**) spectrum. (**J**–**L**) 24 h exposure: (**J**) HAADF, cell wall hole (orange arrow), (**K**) EDX images with Si particles inside empty cells (white arrows), Ca and P (**L**) spectrum. Scale bar, 1 µm. Elemental images for Si, Na, Ca, and P as separate elements are provided in the Supplementary Materials (Figures S1 and S2), as well as an overview of the elemental composition as mass percentages (Supplementary Table S4).

ICP-OES analysis detected substantial Si and Na concentrations alongside a drop in Ca and P compared to RPMI controls in the 2h-BAG-eluate (Figure 1). To further characterize the elemental composition of the deposits observed by SEM on bacterial cells (Figure 7), we next examined the localization of the 2h-BAG-eluate-derived ions and their effects on bacterial cells. STEM-EDX was performed on *S. aureus* exposed for 4 and 24 h to 2h-BAG-eluates to generate spectra of all elements and their relative amounts (based on characteristic peak intensity) present in the sections, while providing 2D spatial distribution images of these elements. The spectra for bacteria exposed for either 4 or 24 h to the 2h-BAG-eluate showed no Na signals associated with the bacterial cells but Ca, P and Si peaks were detected and the element images showed Si, Ca and P on or in association with the cells (Figure 9E,K). P signals were present in both control and 2h-BAG-eluate-exposed samples, likely reflecting the intrinsic natural abundance of P in nucleic acids and phospholipids (Figure 9B,E,H,K and Supplementary Figures S1 and S2). The mass percentage (wt%) of P in the 2h-BAG-eluate-exposed sample (Supplementary Table S4) was not increased compared to the control and therefore does not necessarily indicate the presence of BAG-released phosphorus. Ca signals were present in both control and treated samples; however, slightly increased in bacterial cells exposed for 24 h to 2h-BAG-eluate, compared to controls (Figure 9H,I,K,L, Supplementary Figures S1 and S2). Cells treated with 2h-BAG-eluate showed increased Si signals in the spectra at 4 and at 24 h (Figure 9F,L) and the elemental images showed primarily localization at the cell surface for the 4 h treatment (Figure 9E), while at 24 h, following pronounced structural disruption and perforation of the cells, Si was also detected within bacterial cells which had lost their cytoplasmic content (Figure 9K). Together, STEM-EDX and TEM analyses revealed that exposure to 2h-BAG-eluate prominently caused silica (Si) deposition on the *S. aureus* cell surface at 4 h, progressing to intracellular accumulation by 24 h, coinciding with cell wall breaches, cytoplasmic depletion, and structural breakdown.

## 4. Discussion

Previous studies on the antibacterial mechanism of BAG S53P4 have largely focused on dissolution-mediated physicochemical properties such as local pH, ion release, and osmotic pressure. However, the underlying cellular-level responses have remained largely unexplored. In this study, we provide insight into the antibacterial mechanism by systematically tracing the cascade of events following BAG dissolution. First, we identified and quantified the ions eluated from BAG powder and subsequently examined their fate within Gram-positive bacteria to elucidate how the resulting eluate environment shapes bacterial physiology and leads to cell death.

ICP-OES analysis revealed that BAG eluates exhibited marked Si release, elevated Na and Ca levels, progressive P depletion over time, and complete Mg loss within 2 h. The killing of *S. aureus* by 2h-BAG-eluate occurred rapidly; more than 90% of cells were dead within 30 min for *S. aureus* and 70 min for *B. subtilis*. As recorded in *B. subtilis*, energy stress and a coordinated transcriptional response were evident within 5 min of exposure, characterized by the repression of growth-related biosynthetic pathways and repression and induction of redox and respiratory stress regulons. This early transcriptional response also reflects immediate stress driven by loss of membrane potential and ionic imbalance, in line with the rapid delocalization of MinD, condensation of nucleoid-associated protein RecA and loss of membrane integrity as indicated by SYTOX uptake. After 4 h exposure to 2h-BAG-eluate, EDX analysis identified Si on the bacterial cell surface and a diffuse Ca signal throughout the cytoplasm. By 24 h, severe cell shape deformation was detected, and cell wall disruption was observed. Moreover, holes in the cell wall enabled entry and accumulation of Si in the cell lumen. Calcium levels increased compared to 4 h exposure. Together, these findings describe a stepwise mechanism in which BAG ionic dissolution and elevated alkalinity drive energy stress and metabolic stress of the bacterial cells, causing protein delocalization, global transcriptional reprogramming, membrane disintegration, ribosome delocalization, and cell wall disruption and puncturing. This, in turn, ultimately causes loss of cytosolic content, penetration of Si and further bacterial cell disruption. Ultimately, dead cells are covered and infiltrated with Si.

### 4.1. BAG Dissolution and Multifactorial Bactericidal Process

Elemental analysis of the eluates showed clear evidence of BAG dissolution. The observed rapid Na increase is consistent with alkali-proton exchange, whereas the Si release reflects silicate network dissolution. Even though silicon is more abundant in the S53P4 glass composition, sodium was released into the solution to a much greater extent (Figure 1). This indicates non-stoichiometric dissolution behavior [44,45]. P concentrations decreased rapidly. Ca and P present in RPMI, together with additional P and Ca released from the glass, likely caused rapid supersaturation and calcium phosphate precipitation, reducing both P and Ca in the 2 h eluate [46]. Ca then gradually increased as glass dissolution continued, whereas any additional P released was likely reprecipitated, keeping P levels low. Near complete Mg depletion in the eluates was observed, which might be due to magnesium hydroxide precipitation at alkaline pH [47] and adsorption to silica gel surfaces via electrostatic interactions with Si-O^-^ group [48]. The pH increased to 11.0 within 2 h and then remained stable between 10.6 and 11.0 over 24 h. The antibacterial assay confirmed that BAG eluates exerted potent bactericidal activity against *S. aureus*, with all undiluted eluates collected between 2 and 24 h causing complete eradication. At 2 h, the earliest elution time point tested, the obtained eluate was already very active, even upon dilution. Notably, the early eluates displayed stronger antibacterial activity than 24 h eluates, even though the Na and Ca concentrations continued to rise with longer elution time, and Si increased at least until 4 h. Thus, ion release and elevated pH alone do not fully explain the bactericidal effect but rather support a multifactorial mechanism in which BAG reactivity creates a hostile physicochemical environment marked by high pH, increased ionic strength, and potentially reactive silica species.

Within this framework, we hypothesize that the distribution of silica species in the eluates may contribute to the observed bactericidal differences. Although 2 h and 24 h eluates exhibit similar total Si levels, the 2 h eluate may contain higher proportions of monomeric silicic acid (Si(OH)_4_) or low-molecular-weight siloxomers with abundant surface silanol (Si-OH) groups, which are highly reactive [49]. These species kill bacteria via membrane disruption through silanol-lipoprotein interactions, mechanical stress from nanoscale topography, and reactive oxygen species (ROS) generation [50,51]. Over time, dissolved silicic acid gradually condenses into larger polymeric and potentially colloidal silica species that contain fewer accessible reactive silanol groups per silicon unit [49]. Elevated Na^+^ and Ca^2+^ concentrations in later eluates may further promote aggregation by reducing electrostatic repulsion between silica species, which may explain why the early eluates retain activity even upon dilution, whereas 24 h eluates show reduced potency.

### 4.2. Consequence of 2h-BAG-Eluate Exposure; Early Events

The SYTOX green fluorescence intensity increased early (~5 min) in both species, preceding substantial CFU decline. This supports a model where the 2h-BAG-eluate first disrupts membrane integrity, causing downstream intracellular distress. Similar patterns have been reported for cationic antimicrobial peptides and ion-releasing biomaterials, where SYTOX green uptake correlates with early loss of membrane integrity and is a prelude to cell death [52,53,54]. Time-kill kinetics revealed rapid bactericidal activity of 2h-BAG-eluate against both *S. aureus* and *B. subtilis*, with slight differences in timing, possibly attributable to the cell wall architecture of these bacterial species [18]. These highly similar rapid-killing profiles justify using *B. subtilis* as a model to investigate responses to 2h-BAG-eluate, including the delocalization of marker proteins and transcriptomic changes.

Alongside ionic disturbances, the rapid depletion of Mg in the eluate likely contributes substantially to the bactericidal activity of the 2h-BAG-eluate. ATP-dependent enzymes are essential for DNA repair and protein synthesis [55]; its depletion prevents bacteria from recovering from the membrane damage and enzyme delocalization caused by the 2h-BAG-eluate, thereby intensifying the overall bactericidal effect. The loss of Mg also weakens the cell envelope and likely makes eluate-induced membrane damage and enzyme delocalization more severe. Such membrane disorganization likely inactivates peptidoglycan (PG) synthesis machinery while autolysin activity (N-acetylmuramyl-L-alanine amidases or β-N-acetylglucosaminidases) continues, potentially even increasing. This imbalance causes unchecked PG breakdown, cell wall destabilization, and hole formation [56,57].

Upon 5 min exposure to the 2h-BAG-eluate, transcriptomic data provide molecular support for the early physicochemical and cellular effects observed in this study. The dominant transcriptional signatures were consistent with acute ionic disequilibrium and cell envelope destabilization. Transcriptomic evidence further supports the central role of ionic imbalance, particularly Mg depletion, in BAG-mediated antibacterial activity. Absence of Mg from 2h-BAG-eluate is expected to exacerbate membrane fragility and disturb macromolecular organization, given its importance in stabilizing negatively charged polymers and maintaining ribosomal structure. Moreover, Mg depletion destabilizes ribosomal subunits, impairs translation efficiency, and disrupts membrane integrity due to reduced charge shielding of anionic phospholipids and teichoic acids [40]. The observed repression of growth-associated regulators and purine biosynthesis genes indicates rapid disengagement from growth and biomass production, consistent with the loss of proliferative capacity observed experimentally. The pronounced upregulation of ribosomal protein genes (Table S2) is consistent with acute ribosomal destabilization followed by an emergency maintenance response. Ribosome structure is highly sensitive to intracellular ionic conditions, and rapid membrane depolarization with ion leakage, including Mg loss, is therefore expected to compromise ribosomal stability [58]. Under such conditions, cells transiently increase transcription of ribosomal protein genes to restore ribosomal stoichiometry and preserve translational capacity [59]. The concomitant upregulation of genes involved in tRNA modification and translational fidelity (Table S2) further supports a stress-response mode focused on damage control. Further, activation of cytoplasmic stress-associated sigma factors aligns with the rapid membrane permeabilization and enzyme delocalization of marker proteins MinD and RecA in *B. subtilis*. These observations align mechanistically with transcriptomics work on borosilicate bioactive glass-polymethyl methacrylate cement systems [60], where *S. aureus* RNA-seq was performed after 6 h of direct contact with cement disks. This showed similar ionic stress responses, downregulation of respiratory chain genes and ribosomal stress signatures.

### 4.3. Later Events; Cell Death and Beyond

SEM images showed that 24 h exposure to 2h-BAG-eluate transformed initially smooth, spherical *S. aureus* cocci with intact envelopes into cells exhibiting shape irregularities, surface holes, and particulate deposits. Shape irregularities align with previous SEM observations of S53P4-treated multidrug-resistant bacteria [13].

To elucidate the sequence of ultrastructural events leading to this 24 h damage and capture earlier changes invisible at SEM resolution, we performed TEM for intracellular detail and EDX to map elemental distribution. TEM imaging provided confirmation of progressive damage in *S. aureus*. At 4 h, untreated control cells retained intact cell envelopes and uniform cytoplasmic density, whereas early morphological irregularities with ribosome-free regions were observed in 2h-BAG-eluate treated cells. These changes are consistent with the transcriptome changes observed very early, after 5 min of exposure (Table S2) as discussed. Ribosome-depleted cytoplasmic regions were similar to those observed in *Mycobacterium abscessus* under antibiotic stress [61]. Indeed, the ensuing drastic envelope destruction and cytoplasmic leakage at 24h are consistent with impairment of peptidoglycan synthesis and uncontrolled autolysin activity [62].

EDX analysis demonstrated an association of Ca and Si with the bacterial cells. Ca was observed evenly distributed across the bacterial cell, with higher amounts at 24 h than at 4 h. Because the 2h-BAG-eluate killed the bacteria rapidly, the Ca likely will have diffused passively over time into the dead bacterial cells. Si selectively accumulated on the bacterial surface after 4 h exposure, and by 24 h, was detected as deposits on the cell surface as well as intracellularly in cells that had lost their cytoplasmic content. The preferential cell surface association of Si may have been facilitated by the binding of Ca^2+^ to the highly anionic cell wall polymers of the Gram-positive cell wall, particularly teichoic acids [63,64]. The binding of Ca^2+^ can locally reduce electrostatic repulsion and promote the association of silica species with the cell wall [65,66]. Interestingly, under alkaline conditions such as those created by the elution of ions from BAG, BAG-associated silanol groups may undergo condensation to form silica gel [67]. This condensed silica gel might become locally retained, generating mechanical stress at weakened regions of the cell wall. The coincidence of intracellular Si signals with TEM-detected perforations in the cell envelope supports a model in which Si accumulation might also contribute to focal rupture of the cell wall, potentially exacerbated by arrested peptidoglycan synthesis and ongoing autolytic activity.

In conclusion, this study demonstrates that the antibacterial activity of BAG S53P4 is governed by a dynamic, multi-step mechanism driven by ion release and pH increase. Specifically, we show that (i) the rapid bactericidal activity is not solely explained by pH, (ii) ion release induces progressive membrane destabilization, metabolism disruption, followed by structural damage, and (iii) ultimately leading to bacterial lysis with associated Si deposition on and in dead cells. Importantly, the antibacterial effect was retained in BAG-derived eluates, demonstrating that released ions can act independently of the solid material.

These findings are significant as they provide biological mechanistic insight into how BAG S53P4 exerts antibacterial effects beyond physicochemical explanations. The ability of BAG-derived ions to act at a distance supports their use in clinical settings for targeting bacteria in surrounding tissues. Furthermore, the rapid ion release may contribute to early bacterial control during surgical interventions, reducing the risk of infection. Overall, this work advances the understanding of BAG S53P4 and supports its development in ion-based formulations and in combination with antibiotics for improved infection management.

## Data Availability

The original contributions presented in this study are included in the article/supplementary material. Further inquiries can be directed to the corresponding authors..

## Supplementary Materials

The following supporting information can be downloaded at: https://www.mdpi.com/article/10.3390/jfb17040201/s1. Table S1: Pairwise statistical comparisons of bactericidal activity across all BAG eluate dilutions and elution time. Statistical significance is indicated as nonsignificant (ns) or significant (* *p* ≤ 0.05, ** *p* ≤ 0.01, *** *p* ≤ 0.001, **** *p* ≤ 0.0001), with exact *p* values shown for each comparison (2 vs. 4, 2 vs. 8, 2 vs. 24, 4 vs. 8, 4 vs. 24, 8 vs. 24) at 100%, 50%, 25%, and 12.5% dilutions; Table S2: Differential gene expression following exposure to 2h-BAG-eluate. The table reports BSU locus tags, fold change (FC), *p*-value, gene and operon annotations and fold change values for individual genes within each operon (FC gene 1–FC gene 32); Table S3: Regulon activity following exposure to 2h-BAG-eluate. The table reports regulon identity, number of genes, average (signed) fold change, variability (MAD), statistical significance (*p*-value, FDR), and inferred regulator state (activated or repressed); Table S4: Average mass % of individual elements in samples of cells exposed for 4 or 24 h to RPMI (controls) or 2h-BAG-eluates. The mass % indicates the weight of a specific element relative to the total weight of all detected elements in the sample; Figure S1: Elemental images of *S. aureus* after 4 h of exposure to RPMI medium as control (A–D) or 2h-BAG-eluate (E,F). A and E, Si; B and F, P; C and G, Ca; D and H, Na. Scale bar is 1 µm; Figure S2: Elemental images of *S. aureus* after 24 h of exposure to RPMI medium as control (A–D) or 2h-BAG-eluate (E,F). A and E, Si; B and F, P; C and G, Ca; D and H, Na. Scale bar is 1 µm.

## Abbreviations

The following abbreviations are used in this manuscript:

BAG	Bioactive Glass
ICP-OES	Inductively coupled plasma optical emission spectroscopy
SEM	Scanning Electron Microscopy
TEM	Transmission Electron Microscopy
STEM-EDX	Scanning transmission electron microscopy-energy dispersive X-ray
RPMI	Roswell Park Memorial Institute 1640 medium
