# Supplementary material for: How Bioactive Glass S53P4 Kills Bacteria
Source: J Funct Biomater. 2026 Apr 19;17(4):201. doi: 10.3390/jfb17040201 (PMC13117131; doi:10.3390/jfb17040201)
Supplement: Supplementary file 1 [file jfb-17-00201-s001.zip › Supplementary file.pdf]

# Supplementary materials

| BAG eluate dilutions | Elution time (h) | Summary | *p*-value |
|---|---|---|---|
| 100% | 2 vs 4 | ns | >0.9999 |
| | 2 vs 8 | ns | >0.9999 |
| | 2 vs 24 | ns | >0.9999 |
| | 4 vs 8 | ns | >0.9999 |
| | 4 vs 24 | ns | >0.9999 |
| | 8 vs 24 | ns | >0.9999 |
| 50% | 2 vs 4 | ns | >0.9999 |
| | 2 vs 8 | ns | >0.9999 |
| | 2 vs 24 | * | 0.0276 |
| | 4 vs 8 | ns | >0.9999 |
| | 4 vs 24 | * | 0.0277 |
| | 8 vs 24 | * | 0.0315 |
| 25% | 2 vs 4 | ns | >0.9999 |
| | 2 vs 8 | ns | 0.9882 |
| | 2 vs 24 | *** | 0.0004 |
| | 4 vs 8 | ns | 0.9907 |
| | 4 vs 24 | *** | 0.0005 |
| | 8 vs 24 | ** | 0.0011 |
| 12.5% | 2 vs 4 | ns | 0.4086 |
| | 2 vs 8 | * | 0.0297 |
| | 2 vs 24 | **** | <0.0001 |
| | 4 vs 8 | ns | 0.5309 |
| | 4 vs 24 | *** | 0.0001 |
| | 8 vs 24 | ** | 0.0054 |

**Table S1:** Pairwise statistical comparisons of bactericidal activity across all BAG eluate dilutions and elution time. Statistical significance is indicated as nonsignificant (ns) or significant (*$p \leq .05$, **$p \leq .01$, ***$p \leq .001$, ****$p \leq .0001$), with exact *p* values shown for each comparison (2 vs 4, 2 vs 8, 2 vs 24, 4 vs 8, 4 vs 24, 8 vs 24) at 100%, 50%, 25%, and 12.5% dilutions.

**Table S2** and **Table S3** are provided as two separate excel files

| Average mass% of element | Condition | | | |
|---|---|---|---|---|
| | 4h control | 4h BAG | 24h control | 24h BAG |
| Carbon | 34,74 | 48,98 | 40,89 | 56,49 |
| Nitrogen | 0,81 | 0,76 | 0,90 | 0,93 |
| Oxygen | 5,76 | 3,72 | 6,96 | 3,46 |
| Vanadium | 0,44 | 0,31 | 0,24 | 0,07 |
| Iron | 10,28 | 7,42 | 5,97 | 1,92 |
| Cobalt | 11,25 | 8,20 | 6,60 | 2,24 |
| Copper | 19,16 | 19,29 | 20,11 | 21,60 |
| Osmium | 7,51 | 6,84 | 8,14 | 7,93 |
| Lead | 7,42 | 1,59 | 7,41 | 2,47 |
| Uranium | 1,48 | 1,47 | 1,44 | 1,56 |
| Silicon | 0,06 | 0,27 | 0,04 | 0,31 |
| Phosphorus | 0,41 | 0,34 | 0,45 | 0,33 |
| Chlorine | 0,64 | 0,71 | 0,81 | 0,63 |
| Sodium | 0,01 | 0,01 | 0,01 | 0,01 |
| Magnesium | 0,01 | 0,01 | 0,01 | 0,00 |
| Potassium | 0,00 | 0,00 | 0,00 | 0,00 |
| Calcium | 0,02 | 0,06 | 0,03 | 0,05 |
| | | | | |

**Table S4:** Average mass % of individual elements in samples of cells exposed for 4 or 24 h to RPMI (controls) or 2h-BAG-eluates. The mass % indicates the weight of a specific element relative to the total weight of all detected elements in the sample.

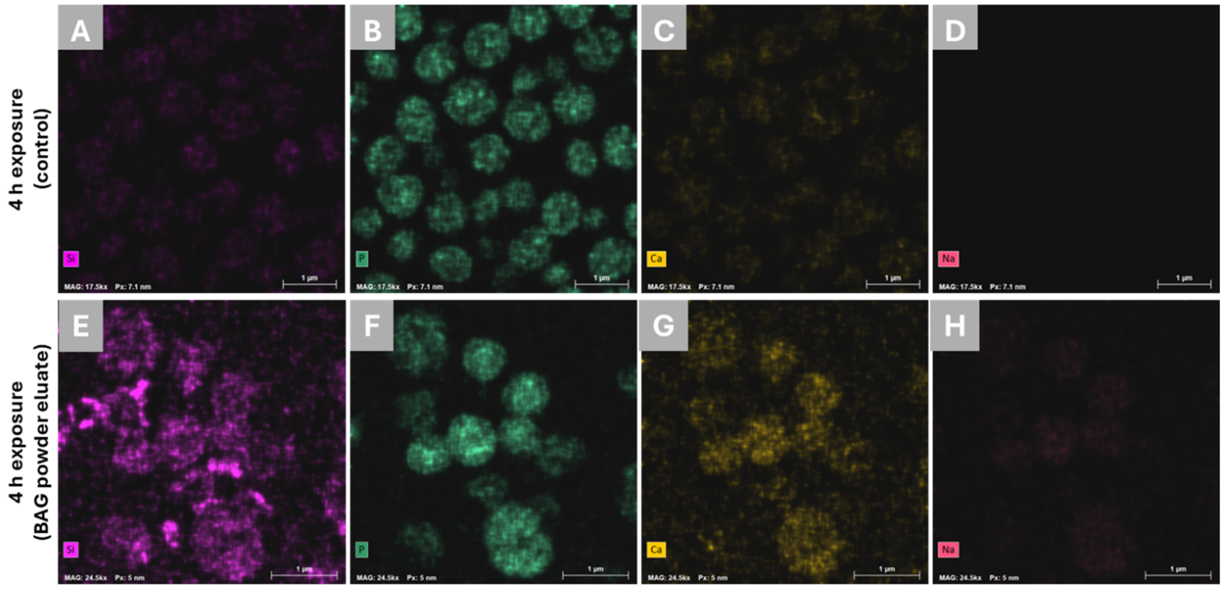


**Figure S1:** Elemental images of *S. aureus* after 4 h of exposure to RPMI medium as control (A - D) or 2h-BAG-eluate (E-F). A and E, Si; B and F, P; C and G, Ca; D and H, Na. Scale bar is 1 µm.

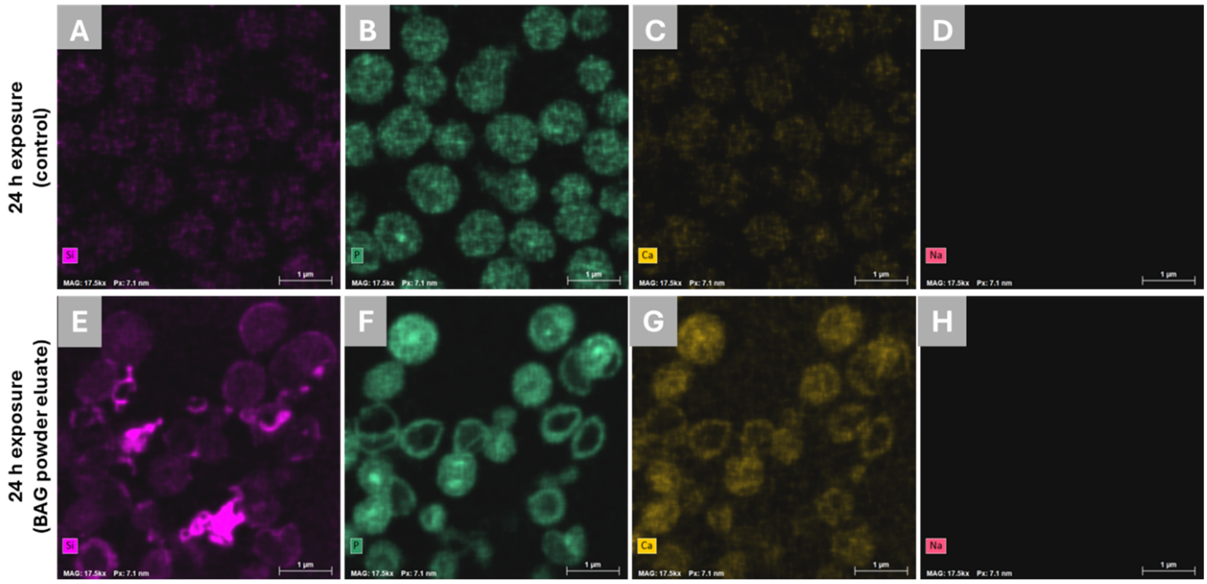


**Figure S2:** Elemental images of *S. aureus* after 24 h of exposure to RPMI medium as control (A - D) or 2h-BAG-eluate (E-F). A and E, Si; B and F, P; C and G, Ca; D and H, Na. Scale bar is 1 µm.
